# Prevalence of Middle East Respiratory Syndrome Coronavirus in Dromedary Camels, Tunisia

**DOI:** 10.3201/eid2707.204873

**Published:** 2021-07

**Authors:** Simone Eckstein, Rosina Ehmann, Abderraouf Gritli, Houcine Ben Yahia, Manuel Diehl, Roman Wölfel, Mohamed Ben Rhaiem, Kilian Stoecker, Susann Handrick, Mohamed Ben Moussa

**Affiliations:** Bundeswehr Institute of Microbiology, Munich, Germany (S. Eckstein, R. Ehmann, M. Diehl, R. Wölfel, K. Stoecker, S. Handrick);; Ministry of National Defense, General Directorate of Military Health, Veterinary Service, Tunis, Tunisia (A. Gritli, H. Ben Yahia, M. Ben Rhaiem);; Military Hospital of Instruction of Tunis Department of Virology, Tunis (M. Ben Moussa)

**Keywords:** MERS-CoV, Middle East respiratory coronavirus, seroprevalence, phylogenetic analyses, dromedary camels, *Camelus dromedarius*, North Africa, Tunisia, viruses, zoonoses, respiratory infections

## Abstract

Free-roaming camels, especially those crossing national borders, pose a high risk for spreading Middle East respiratory syndrome coronavirus (MERS-CoV). To prevent outbreaks, active surveillance is necessary. We found that a high percentage of dromedaries in Tunisia are MERS-CoV seropositive (80.4%) or actively infected (19.8%), indicating extensive MERS-CoV circulation in Northern Africa.

Middle East respiratory syndrome (MERS) coronavirus (MERS-CoV) has the highest lethality of all know human coronaviruses; the case-fatality rate is 34.3% (*1*). The virus, first isolated in Saudi Arabia in 2012 (*2*), most likely originates from bats (*3*). However, several studies suggest that the zoonosis is mainly transmitted to humans by dromedary camels (*Camelus dromedarius*) (*4*–*6*).

As of January 2020, a total of 2,519 human MERS-CoV infections and 866 related deaths had been reported to the World Health Organization from 27 countries. Most of these cases (84.2%), including 788 related deaths, occurred in Saudi Arabia (*1*).

Given the close trading links between the Middle East and Africa, the risk for transferring the zoonosis is high. Tunisia is not a popular trading location, which makes undocumented transfers of dromedary camels within the country or with neighboring countries difficult to track. Only 3 human MERS cases have been imported from Qatar (*7*), and no autochthonous MERS-CoV infections have been reported for Tunisia. However, severe underestimation of human MERS cases is highly probable because of the broad range of manifestations, from asymptomatic infection to acute pneumonia.

Furthermore, epidemiologic surveillance of MERS-CoV is limited in Tunisia, and no respective data for neighboring countries is publicly available. Two studies analyzing MERS-CoV prevalence in dromedaries in Tunisia reported high seropositivity of the sampled animals (49.0% and 87.3%) (*8*,*9*). However, those studies analyzed dromedary camels from livestock markets, slaughterhouses, and meat farms, without representing natural habitats. This limitation complicates drawing realistic conclusions about geographic and age-dependent distributions. We investigated the prevalence of MERS-CoV in dromedary camels in Tunisia primarily by sampling animals that roam freely through the desert during summer to determine an authentic representation of the distribution pattern.

## The Study

Winter is mating and birthing season for dromedary camels. Therefore, animals kept for milk and meat production gather in areas that provide access to salty plants and other minerals. This environment provides an optimal opportunity to catch and examine large numbers of animals from different herds and origins, given that the camels that roam freely through the desert the rest of the year congregate simultaneously.

**Figure 1:**
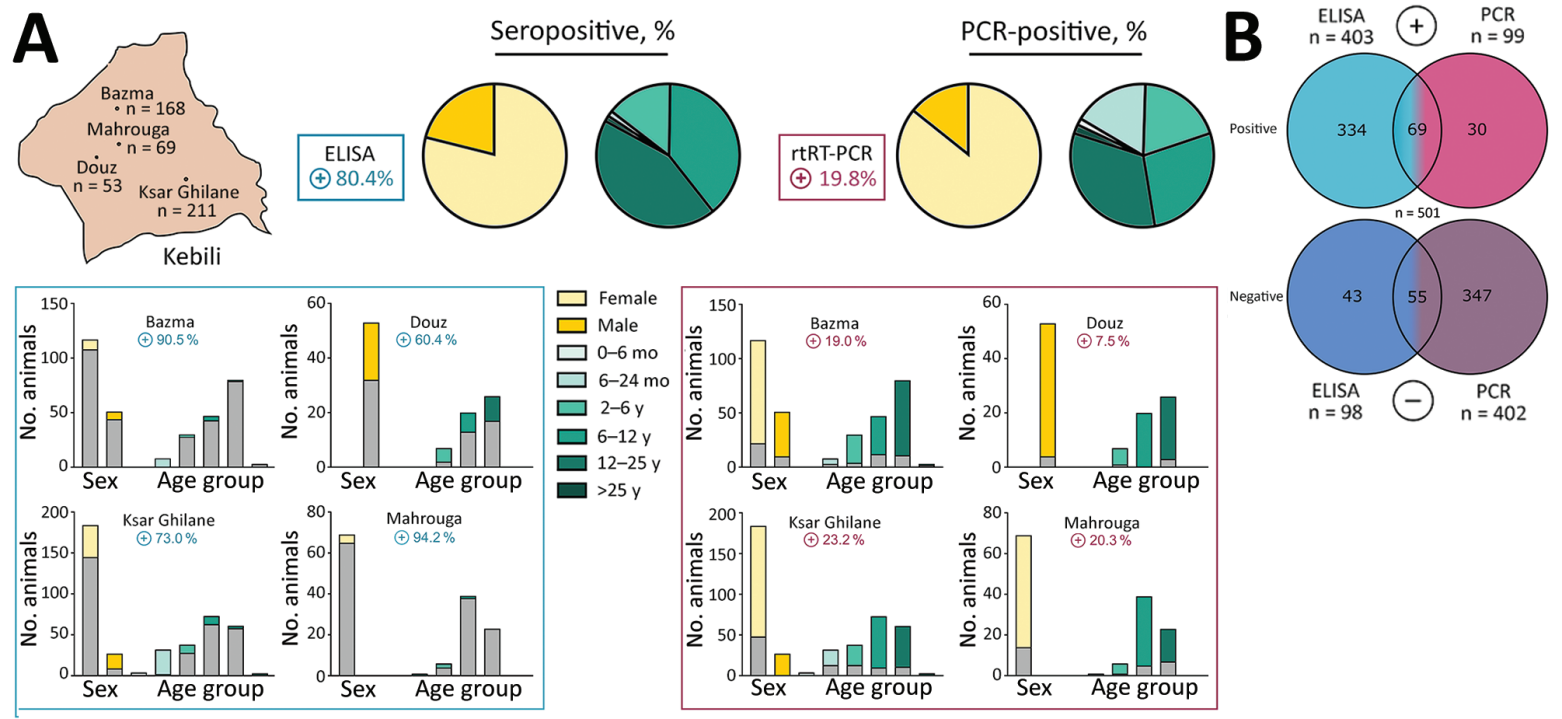
MERS-CoV prevalence in dromedary camels, Tunisia. A) Overview of seropositive and PCR-positive dromedaries from 4 different sampling areas (Appendix), by sex and age group. Blues boxes represent seropositive dromedaries and purple boxes PCR-positive dromedaries. Bars in chart represent total number of sampled animals; gray-shaded bar sections represent total number of MERS-CoV–positive animals. In the Douz area, all dromedary camels sampled were used for patrol or transport purposes and were exclusively adult male animals. B) Distribution of ELISA (blue) and PCR (purple) results, by number. Bright colors represent positive results, dark colors negative results; 2-colored areas represent animals that are either positive for both ELISA and PCR or negative for both. MERS-CoV, Middle East respiratory syndrome coronavirus.

In January 2020, we collected serum samples and nasal swabs of 382 gathered animals in Tunisia. We sampled an additional 119 camels used for transport or patrol purposes, all of which were males and kept enclosed. The specimens were collected from 20 different locations within the Kebili Governorate (Table; Figure 1, panel A). Furthermore, serum samples of 22 camel keepers and 2 veterinarians were obtained. We have compiled details of our sampling and testing methods (Appendix).

**Table:** MERS-CoV IgG seropositivity and viral RNA presence in dromedary camels, by selected sampling parameters, Tunisia*

Sampling parameter	No. dromedaries	ELISA serologic testing, no. (%) positive for MERS-CoV IgG	Molecular biology rRT-PCR, no. (%) positive for MERS-CoV RNA
Sex			
M	131	86 (65.6)	14 (10.7)
F	370	317 (85.7)	85 (23.0)
p value		<0.05	<0.01
Age group			
Juvenile	45	2 (4.4)	18 (40.0)
0–6 mo	4	0 (0)	1 (25.0)
6–24 mo	41	2 (4.9)	17 (41.5)
Adult	456	401 (87.9)	81 (17.8)
2–6 y	81	62 (76.5)	19 (23.5)
6–12 y	179	157 (87.7)	28 (15.6)
12–25 y	190	176 (87.9)	32 (16.8)
>25 y	6	6 (100)	2 (33.3)
p value, juvenile compared with adult		<0.00001	<0.01
Sampling site			
Ksar Ghilane, n = 6	211	154 (73.0)	49 (23.2)
Site 1	28	20 (71.4)	7 (25.0)
Site 2	20	8 (40.0)	0 (0)
Site 3	30	26 (86.7)	6 (20.0)
Site 4	20	19 (95.0)	1 (5.0)
Site 5	73	50 (68.5)	25 (34.2)
Site 6	40	31 (77.5)	10 (25.0)
Bazma, n = 7	168	152 (90.5)	32 (19.1)
Site 1	25	24 (96.0)	2 (8.0)
Site 2	30	25 (83.3)	8 (26.7)
Site 3	15	13 (86.7)	3 (20.0)
Site 4	15	14 (93.3)	1 (6.7)
Site 5	21	20 (95.2)	4 (19.0)
Site 6	16	13 (81.3)	6 (37.5)
Site 7	46	43 (93.5)	8 (17.4)
Douz, n = 5	53	32 (60.4)	4 (7.5)
Site 1a	4	3 (75.0)	0 (0)
Site 1b	4	0 (0)	0 (0)
Site 2	24	18 (75.0)	3 (12.5)
Site 3	18	10 (55.6)	1 (5.6)
Site 4	3	1 (33.3)	0 (0)
Mahrouga, n = 2	69	65 (94.2)	14 (20.3)
Site 1	42	40 (95.2)	3 (7.1)
Site 2	27	25 (92.6)	11 (40.7)
p value for comparisons among all 4 main sites		0.05	Not significant
Total	501	403 (80.4)	99 (19.8)

*MERS-CoV, Middle East respiratory syndrome coronavirus; rRT-PCR, real-time reverse transcription PCR.

We analyzed all 501 dromedary serum samples for MERS-CoV–specific antibodies by ELISA and found 80.4% to be seropositive for MERS-CoV IgG. At 85.7%, MERS-CoV seropositivity was higher in female than in male camels (65.6%) (Table; Figure 1, panel A).

Although none of the calves (0–6 months of age) and 4.9% of the juvenile camels (6–24 months of age) were seropositive for antibodies against MERS-CoV, relative seropositivity increased with age (Table). None of the camel keepers or veterinarians was seropositive, indicating no previous MERS-CoV infection.

Screening the dromedary nasal swab specimens for active virus infections with real-time reverse transcription PCR revealed MERS-CoV RNA in 19.8%. However, cycle thresholds >30 (for all but 6 samples) indicated low virus concentrations. Female animals (23.0%) actively shed MERS-CoV RNA, whereas only 10.7% of the male camel specimens were PCR-positive. In contrast to the immunologic findings, a high percentage (40%) of juvenile camels (<2 years of age) shed MERS-CoV RNA, compared with 17.8% of the adult camels that tested positive (Table, Figure 1, panel A)

In summary, 433 of 501 dromedaries tested positive for MERS-CoV. A total of 334 animals were seropositive for MERS-CoV IgG but did not shed MERS-CoV RNA. Of these, 30 dromedary swab specimens contained MERS-CoV RNA, but no specific antibodies were found in the respective serum samples. Sixty-nine PCR-positive dromedary camels also had MERS-CoV antibodies, indicating reinfection (Figure 1, panel B).

**Figure 2:**
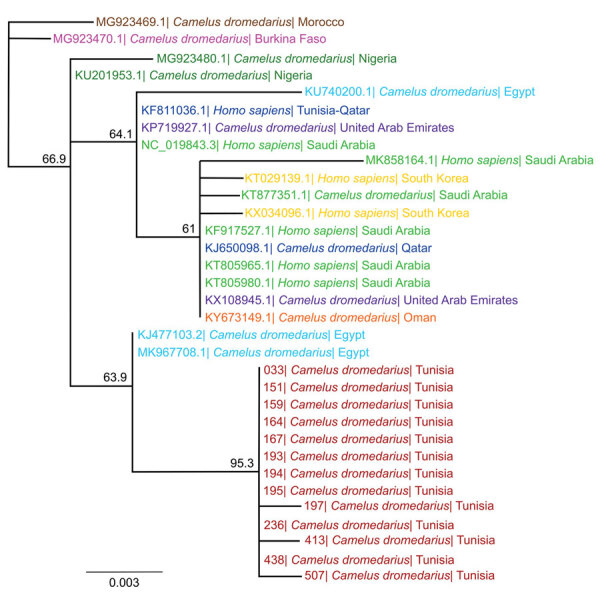
Phylogenetic analysis of MERS-CoV samples from dromedary camels in Tunisia, conducted by using the spike RBD. We used 720-bp fragments of the MERS-CoV spike RBD amplified from nasal swab samples of 13 dromedary camels and published RBD sequences of representative MERS-CoV strains from other countries to create the phylogenetic tree using Geneious Prime Tree Builder (Geneious Biologics, https://www.geneious.com). Branches are shaded by country: red represents sequences from Tunisia (this study); brown represents Morocco, pink Burkina Faso, dark green Nigeria, blue Egypt, dark blue Qatar, green Saudi Arabia, yellow South Korea, purple United Arab Emirates, and orange Oman. GenBank accession numbers are provided for reference sequences. Numbers indicate bootstrap values (1,000 pseudo-replicates). Scale bar indicates sequence divergence (% nucleotide substitutions). MERS-CoV, Middle East respiratory syndrome coronavirus; RBD, receptor-binding protein.

Attempts to cultivate the virus from all respective PCR-positive swab specimens were unsuccessful, most likely because of the low virus concentrations in the samples. Presumably, whole-genome sequencing did not work for the same reasons. However, we performed Sanger sequencing of cDNAs obtained from PCR-positive samples with the highest viral concentrations and subsequently conducted phylogenetic analysis with a 720-bp fragment of the spike receptor-binding protein. The analyzed nucleotide sequences from the dromedaries in Tunisia differ from previously published MERS-CoV sequences and therefore form a separate group distinct from strains found in Arabia. Two MERS-CoV isolates in Egypt, however, cluster in the same clade (Figure 2).

## Conclusions

On the continent of Africa, active surveillance, longitudinal studies, and epidemiologic monitoring are scarce, and little is known about the prevalence and circulation of MERS-CoV in many regions. Whether MERS-CoV lineages in Africa have a lower tendency to cross the species barrier and infect humans is not fully understood. Therefore, closing the gaps in surveillance and virus prevalence data remains a focus for all regions with dromedary camel populations.

Seroprevalence studies in Egypt, Ethiopia, Nigeria, and Kenya all indicate MERS-CoV circulation within camel herds, reporting seropositivity rates ranging from 30% to 100% (*10*). For dromedaries in Tunisia, only 2 studies have been published, reporting 49% and 87.3% MERS-CoV seropositive animals and only 0.7% active viral shed (*8*,*9*).

However, most studies focus on locations where large numbers of camels congregate (e.g., abattoirs, large-scale farms, harbors, or livestock markets). At these locations, dromedaries are kept at a substantially increased population density compared with their normal habitats. This practice, referred to as crowding, increases stress for individual animals (*11*). Under these circumstances, increased intensive animal contact can lead to higher transmission rates of various microorganisms. Crowding, in combination with animal transport, is known to promote infections of the upper and lower respiratory tract, especially in cattle (*12*). In contrast, the camels investigated in our study represent a rare example of MERS-CoV prevalence in animal groups with a natural herd structure in northern Africa.

We found extensive MERS-CoV IgG seropositivity (80.4%) and high ratios of MERS-CoV RNA (19.8%) among dromedaries in Tunisia. Compared with adult animals, juvenile camels were more likely to have active MERS-CoV infections and less MERS-CoV IgG in their serum samples. Furthermore, some dromedaries appeared to have MERS-CoV reinfections, explained by the fact that coronaviruses tend to establish endemic infection patterns with high seroprevalence and low but continuous viral shedding in their natural host (*13*). Waning antibodies in combination with antigenic drift of the virus fosters reinfection events (*14*).

A limitation of our study is that the low sample size of humans tested, comprising 22 camel keepers and 2 veterinarians (data not shown), precludes drawing generalized conclusions. Furthermore, no nasal swab specimens were collected from camel keepers to check for active MERS infections. Also, no phenotypic or whole-genome analysis of MERS-CoV strains from the dromedary camels was possible because virus growth and next-generation sequencing were not successful because of low viral concentrations.

In conclusion, the high seroprevalence of MERS-CoV antibodies and the active shed of MERS-CoV RNA indicate the widespread nature of the virus in dromedaries in Tunisia. However, more extensive studies in the human and dromedary camel populations and in-depth whole-genome sequence analysis of circulating MERS-CoV strains are required to increase epidemiologic understanding of the disease and its infection dynamics.

Appendix Additional information about the prevalence of Middle East respiratory coronavirus in dromedary camels, Tunisia.
