# Supplementary material for: Prevalence of Middle East Respiratory Syndrome Coronavirus in Dromedary Camels, Tunisia
Source: Emerg Infect Dis. 2021 Jul;27(7):1964–8. doi: 10.3201/eid2707.204873 (PMC8237907; doi:10.3201/eid2707.204873)
Supplement: Appendix — Additional information about the prevalence of Middle East respiratory coronavirus in dromedary camels, Tunisia. [file 20-4873-Techapp-s1.pdf]

Article DOI: https://doi.org/10.3201/eid2707.204873

Prevalence of Middle East Respiratory Syndrome Coronavirus in Dromedary Camels, Tunisia

Appendix

Materials and Methods

Sampling

A total of 501 dromedary camels' nasal swabs as well as serum samples were collected at 20 different sites for Middle East Respiratory Syndrome Coronavirus (MERS-CoV) screening in January 2020. The individual sampling sites were in the surrounding areas of Ksar Ghilane (6 sites; n = 211), Douz (5 sites; n = 53), Bazma (7 sites; n = 168) and Mahrouga (2 sites; n = 69) within the Kebili governorate (Table 1, Figure 1A,). 382/501 samples were obtained from dromedary camels in their natural habitat at Ksar Ghilane (sampling site 1 and 3–6,) Bazma (sites 1–6) and Mahrouga (sites 1+2). Those herds kept for milk and/or meat production each consisted of one sultan, its female harem and some juvenile male animals. However, to avoid too high stress level for the animals, not all camels in a herd could be sampled. The remaining 119 camels, exclusively adult males used for transport or patrol purposes and therefore kept enclosed, were from around Douz (sites 1a+b-4), Ksar Ghilane (site 2) and Bazma (site 7).

A total of 131 male and 370 female dromedary camels ranging in age from juvenile (0–6 months: 4 camels and 6–24 months: 41) to adult (2–6 years: 81 camels; 6–12 years: 179; 12–25 years: 190 and >25 years: 6) were sampled.

Additionally, 22 camel keepers (men between the ages of 12 to 69) from the sampling sites in Ksar Ghilane and Douz) having daily contact with their dromedary camels as well as two veterinaries (men, 25 and 48 years) with frequent animal contact were willing to provide serum for investigation of previous MERS-CoV infections.

Immunoserological testing

Seroprevalence of anti-MERS-CoV IgG in dromedary camels as well as camel keepers was investigated by performing indirect ELISA assays (Euroimmun AG, Germany) according to the manufacturer's protocol.

Molecular testing

Viral RNA was extracted using the QIAamp Viral RNA Mini Kit (QIAGEN, Netherlands). Subsequently, real-time reverse transcription PCR (rtRT-PCR) was performed targeting the genetic region upstream of the *E* gene (*upE*) (for screening) and *ORF1a* (for confirmation), respectively, as described elsewhere (*1*,*2*).

Statistical analysis

Associations between MERS-CoV prevalence in dromedary camels and the study parameters (sex, age and sampling site) were analyzed by Pearson Chi-square test for comparison of categorical data. At a p-value of less than 0.05, statistical significance was considered.

Virus isolation in cell culture

Isolation of live virus was attempted on Vero E6 cells under BSL3 conditions. Briefly, 250 µl of virus transport medium of MERS-CoV rtRT-PCR-positive nasal swabs was used as inoculum for cell cultures in T25 flasks. Cells were washed with serum free medium, inoculated with sample fluid and incubated for one hour. Afterwards, the inoculum was removed and the cellular layer was washed three times. The final culture medium contained MEM with 2% FBS and 5x antibiotic-antimycotic additive (GIBCO, USA). Cells were incubated for 7 days with one subsequent subpassage of supernatant on fresh cells. Cultures were regularly screened for cytopathic effects and supernatant was screened for an increase in MERS-CoV RNA.

Genomic spike RBD amplification

For phylogenetic analysis a partial 774-bp fragment of the spike (*s*) gene containing the RBD was amplified by running a rtRT-PCR (SSIII qRT-Kit, Invitrogen, United States) using the primer pair pre-RBD fwd (GAATCTGGAGTTTATTCAGTTTCGT) and pre-RBD rev (ACGGCCCGAAACACCATAG) (*3*). Subsequently, spike RBD (720-bp) was amplified by performing a nested PCR with the primers RBD fwd (GAAGCAAAACCTTCTGGCT) + RBD rev (ATATTCCACGCA) using Q5 HotStart MasterMix Kit (New England Biolabs, USA) with

the respective pre-fragment as DNA template. The resulting PCR product was gel-purified (Mini kit for gel extraction and PCR clean up, Macherey-Nagel, Germany) and sequenced with the latter primers (Eurofins Genomics Germany GmbH, Germany).

Sequence analysis

The phylogenetic tree was constructed based on 13 *s* RBD (GenBank AcNo: MW322770-MW322782) sequences, obtained from rtRT-PCR-positive dromedary specimens with the highest viral amounts and representative MERS-CoV sequences from different countries using Geneious Prime (Version 2020 1.2) by applying the neighbor-joining method with Tamura-Nei's genetic distance model and 1,000 bootstrap replicates.

Ethics Statement

The use of the biologic material described in the underlying study was approved by the Ethics Committee of the Military Hospital in Tunis (Decision N° 57/2020/CLPP) and written consent forms were signed by all included participants.

References

1. Corman VM, Eckerle I, Bleicker T, Zaki A, Landt O, Eschbach-Bludau M, et al. Detection of a novel human coronavirus by real-time reverse-transcription polymerase chain reaction. Euro Surveill. 2012;17:20285. PubMed https://doi.org/10.2807/ese.17.39.20285-en

2. Corman VM, Müller MA, Costabel U, Timm J, Binger T, Meyer B, et al. Assays for laboratory confirmation of novel human coronavirus (hCoV-EMC) infections. Euro Surveill. 2012;17:20334. PubMed https://doi.org/10.2807/ese.17.49.20334-en

3. Kandeil A, Gomaa M, Nageh A, Shehata MM, Kayed AE, Sabir JSM, et al. Middle East respiratory syndrome coronavirus (MERS-CoV) in dromedary camels in Africa and Middle East. Viruses. 2019;11:717. PubMed https://doi.org/10.3390/v11080717
